# Implantation of the Generative Glands and Its Therapeutic Possibilities

**Published:** 1915-03

**Authors:** G. Frank Lydston

**Affiliations:** Formerly Professor of Genitourinary Diseases and Syphilology, Medical Department, State University of Illinois


### Abstracts and Selections.

### Implantation of the Generative Glands and Its Therapeutic Possibilities.

BY G. FRANK LYDSTON, M. D., FORMERLY PROFESSOR OF GENITO-URINARY DISEASES AND SYPHILOLOGY, MEDICAL DEPARTMENT, STATE UNIVERSITY OF ILLINOIS.

A REVIEW BY Z. T. SCOTT, M. D., AUSTIN, TEXAS.

At the request of the editor of the TEXAS MEDICAL JOURNAL I have reviewed, and in the following brief form shall attempt to condense the article on "Implantation of the Generative Glands and Its Therapeutic Possibilities" by Dr. G. Frank Lydston of Chicago. This article originally embraces 93 pages and is reprinted from the *New York Medical Journal* for October 17, 24, 31, and November 7, 1914.

In his opening remarks, Dr. Lydston very rightfully refers to the "Oophorectomy Craze" as a dark blot upon the history of surgery in the nineteenth century,—and it would be well to add that the blot is still moist now on the eve of the twentieth century. Naturally the supreme effort of the conscientious surgeon would be to supplement deficient generative glands or replace those, the remorse of which was made necessary through disease. Dr. Lydston relates the experiments of Bardin, Berthold, Johannes Muller, Herlitzka, Lode, Ribbert, Guthrie, Cloud Bernard, Biedl and the present hormone period and the period of aseptic and successful transplantation of organs. The work of Carrell is cited as the precursor of greater events in auto-transplantation of the testis may be done either with or without anastomosis, the sevatune or adjacent tissues being the favored site. For the ovary the sites are preferable in the following order: the peritoneal space, 1, 2; the cul-de-sac of Douglas, 3; the labia majora, 4; beneath the mammary gland, 5; the pubic region, and 6 the rectus muscle.

Tuffier asserts that transplants from black to white and vice versa fail. Dr. Lydston is unable to confirm or deny this. Dr. Lydston proceeds to give the technic of transplantation, which is simple, using either the fresh specimen or specimens removed hours after death. He reports many experiments on various classes of patients and gives an interesting review of the individual cases, including the varied physiological changes incident to the operation. These embrace modifications in every function, practically in the body. His conclusions are valuable and are given verbatim:

1. At least temporarily, probably permanently—and indubitably therapeutically—successful total or partial implantation of human sex glands in both male and female is practicable.

2. Glands taken from the living subject are most desirable, though rarely obtainable. The closer the blood relationship of donor and recipient the better, but such relationship is not necessary for purely therapeutic purposes.

3. Judging by my own auto-experiments and hetero-experiments, and with due respect to Carrell's observations, I conclude that, while glands frozen before decomposition may be available, they must be used without freezing and very promptly after removal from the body, to obtain a fair average of successes. Glands taken from the healthy dead body at any time prior to the beginning of decomposition are of therapeutic value equal to that of those taken in vivo, if implantation succeeds. Portions of glands are to a certain degree therapeutically serviceable according to conditions and dose.

4. Where we are not warranted in incurring risk the subject from which the glands are taken should be selected with extreme care.

5. The ovary and the testis probably are alike in their susceptibility to implantation, both from the living to the living and from the dead to the living. If any difference exists it seemingly is in favor of the ovary. In human beings the glands of one sex is transplantable upon the other, and it is possible that the hormone of the one is useful to the other. My experiments apparently show that the tissues of the female are more hospitable to the implanted male sex glands than are the tissues of the male.

6. The benefits of implantation probably accrue irrespective of the site of the implantation, but the vicinity of the peritoneum (extra-abdominal) in the female, and of the tunica vaginalis in the male, are the sites of election.

7. The internal sex gland secretion is stimulant, nutrient, tonic, and reconstructive, and should increase resistance to disease. Certain chronic infections, notably tuberculosis, serious anemia, neurasthenia, and conditions of profound debility should be benefited by implantation.

8. The development of senility possibly can be retarded and longevity increased by internal sex secretion derived from implantation. The climacteric may be postponed by it, or the disagreeable features of the climacteric relieved.

9. Used at a very early period in the disease, internal sex secretion should theoretically be the logical remedy for dementia praecox and allied conditions.

10. The internal sex gland secretions via implantation has a very useful field in the treatment of impotence in the male.

11. Implantation, with or without anastomosis in the male, possibly may have a certain range of usefulness in sterility in both sexes.

12. Defective and aberrant physical or psychical sex development and differentiation, inversions and perversions, are definite indications for sex gland implantation. Certain cases of cryptorchidism and imperfect testicular development are an especially promising field for it.

13. Chronic diseases of the skin due to, or modified by nutritional disturbances, notably certain types of chronic eczema, psoriasis and ichthyosis—in a certain proportion of cases—apparently are likely to be benefited and possibly cured by sex gland implantation.

14. That arteriosclerosis will in its early stages be benefited by sex gland implantation is probable. Inferentially, if taken early, senile dementia possibly may show beneficial results.

15. All conditions incidental to sex gland mutilations in either sex afford a positive indication for sex gland implantation, the probability of benefit being inversely as the length of time that has elapsed since the mutilation, and dependent on the age at which it occurred.

16. The most important point of all is that, in properly selected cases, successful implantation ought inevitably to increase physiological efficiency, with all the benefits accruing therefrom. With increased physiological efficiency come individual and social efficiency.

17. Opportunities should be sought in the human subject for histological study of implanted glands at varying periods after implantation, to determine in what degree both generative and internal secretion gland tissues endure.

18. Every effort should be made so to amend our laws that viable tissues of all kinds, notably internal secretory glands, shall become available to science. To this end the public especially should be made to understand that the sacrifice of a portion of thyroid or of a single ovary or testis by a living subject is not disastrous. The author believes that possibly there are times when such a sacrifice would restore reason, perhaps even save life. Legislation and public sentiment should favor scientific research. Between the anti-vivisectionists, on the one hand, and popular reverence for the dead human body, on the other, we are in sore straits. Why should there be a waste of material which, if properly used, possibly might add so much to the health, happiness, efficiency, and even to the longevity of the human race? Let us strive for the conservation of biological energy.

As matters now stand only persons in affluent circumstances and very few even of these, and a limited number of the poor in our institutions can avail themselves of the sex gland implantation.

### APPENDIX.

Since my first six experiments were made and the greater part of this paper was written, the author's attention has been called to some very interesting observations which, so far as they go, serve to strengthen the position herein recorded regarding the value of sex gland transplantation hormone therapy.

Leopold-Levi (43) reports a case of rheumatism and psoriasis, associated with hypothyroidism, successfully, treated with thyroid extract. In another case of psoriasis excellent results followed the administration of "testicular powders."

Garre of Bonn has expressed himself enthusiastically over the prospects of thyroid implantation. He says:

Transplantation of the thyroid will revolutionize the work of the social worker within a few years. Crime, idiocy, the lack of development in children, degeneracy will be lessened through the knowledge of this remarkable organ which is just drawing upon us.

To the thyroid have been traced thousands upon thousands of cases of stunted growth, of mental undevelopment of idiocy, and such defects. An undeveloped thyroid means an undeveloped child.

Let us take the case of father and son. The father has a normal thyroid, and the son's is undeveloped, hence he is making no progress, mentally or physically. We can remove one-third or even two-thirds of the father's gland without injuring him in the least, and by transplanting this to the son can soon bring him to positive normal development.

Transplantation of thyroid from the dead to the living under proper conditions probably is quite as practicable as transplantation from the living to the living as Carrell has stated, and Garre seems to believe, and contrary to my experience with the testicle, donor and recipient absolutely must be closely related.

Bandler (44) states that he has used ovarian extract with success in dysmenorrhea, the disturbances of the climacteric, atrophy of the uterus, and amenorrhea. As he usually combines iron with the ovarian extract, comment is unnecessary.

Dubois and Boulet (45) assert that intravenous injections of prostatic extract produce a fall of blood pressure with an associated increase of brain volume and a lessening of renal tissue volume.

Iscovesco (46) notes the effects of a "lipoid" extracted from the testis and ovary. Clinical trial of this lipoid in the daily dose of 0.02 gram (one-third of a grain) for thirty days in eleven patients suffering from hypochondria, or neurasthenia with sexual weakness and in eight aged men, resulted in increased general vigor, a better mental attitude, and improved capacity for work. In four of the eight old men the blood pressure was lowered. Vesical tenesmus, due to prostatic hypertrophy in three of the cases, disappeared completely and permanently after an injection of 0.16 gram (two and a half grains) of the liquid. No toxic effects were noted from the large doses, either in these patients or in the animals. The author lays stress upon the erythrocytic properties of the lipoid and extols it in the treatment of severe anemia, notably chlorosis, and in severe conditions of innutrition.

